# Immunity and Viral Infections: Modulating Antiviral Response via CRISPR–Cas Systems

**DOI:** 10.3390/v13071373

**Published:** 2021-07-15

**Authors:** Sergey Brezgin, Anastasiya Kostyusheva, Ekaterina Bayurova, Elena Volchkova, Vladimir Gegechkori, Ilya Gordeychuk, Dieter Glebe, Dmitry Kostyushev, Vladimir Chulanov

**Affiliations:** 1 National Medical Research Center of Tuberculosis and Infectious Diseases, Ministry of Health, 127994 Moscow, Russia; seegez@mail.ru (S.B.); ak@rcvh.ru (A.K.); vladimir@chulanov.ru (V.C.); 2 Institute of Immunology, Federal Medical Biological Agency, 115522 Moscow, Russia; 3 Scientific Center for Genetics and Life Sciences, Division of Biotechnology, Sirius University of Science and Technology, 354340 Sochi, Russia; 4 Chumakov Federal Scientific Center for Research and Development of Immune-and-Biological Products of Russian Academy of Sciences, 108819 Moscow, Russia; 79153645941@ya.ru (E.B.); gordeychuk_iv@chumakovs.su (I.G.); 5 Department of Infectious Diseases, Sechenov University, 119991 Moscow, Russia; az@rcvh.ru; 6 Department of Pharmaceutical and Toxicological Chemistry, Sechenov University, 119991 Moscow, Russia; mr.faul@mail.ru; 7 Department of Organization and Technology of Immunobiological Drugs, Sechenov University, 119991 Moscow, Russia; 8 National Reference Center for Hepatitis B Viruses and Hepatitis D Viruses, Institute of Medical Virology, Justus Liebig University of Giessen, 35392 Giessen, Germany; Dieter.Glebe@viro.med.uni-giessen.de

**Keywords:** CRISPR/Cas, interferon effector proteins, interferon induction, pathogen recognition receptor, pathogen-associated molecular pattern, Toll-like receptor, cGAS/STING, DNA sensors, interferon stimulated genes, pooled libraries, epitranscriptomics, HBV, HDV, HCV, HIV, SARS-CoV-2, yellow fever virus, KSHV, HSV, EBOV, ZIKV, influenza A virus, CHIKV

## Abstract

Viral infections cause a variety of acute and chronic human diseases, sometimes resulting in small local outbreaks, or in some cases spreading across the globe and leading to global pandemics. Understanding and exploiting virus–host interactions is instrumental for identifying host factors involved in viral replication, developing effective antiviral agents, and mitigating the severity of virus-borne infectious diseases. The diversity of CRISPR systems and CRISPR-based tools enables the specific modulation of innate immune responses and has contributed impressively to the fields of virology and immunology in a very short time. In this review, we describe the most recent advances in the use of CRISPR systems for basic and translational studies of virus–host interactions.

## 1. Introduction

The development of antibacterial drugs in the first half of the 20th century provided the opportunity to control the most serious bacterial infections and markedly reduced the corresponding fatality and disability rates. However, compared to bacteria, viruses have a far more complicated life cycle and are remarkably more heterogeneous, which significantly complicates the development of broad-spectrum antivirals. The pandemic caused by the novel SARS-CoV-2 coronavirus has caused millions of deaths worldwide and resulted in the most severe economic recession since World War II [1]. Chronic viral infections by pathogens such as the human immunodeficiency virus (HIV) and the hepatitis B, C, and D viruses (HBV, HCV, and HDV, respectively) have been causing epidemics that kill millions of people for many decades [2,3]. For most viral diseases, there are no effective antiviral therapies or no therapies at all. A different approach is to activate specific or non-specific immune responses (immunotherapy or immunomodulation), which can contribute to the elimination or cessation of viral replication by adaptive and/or innate immunity.

The most important components of antiviral immune defense include innate immunity (mostly the interferon system [4] and natural killer cells [5]) and adaptive immunity (CD4+ and CD8+ T cells). Compared to CD8+ T-cytotoxic lymphocytes [6] and natural killer (NK) cells [7], which can destroy infected cells and thus contribute to viral clearance, activation of the interferon (IFN) system can upregulate expression of interferon-stimulated genes (ISGs), which directly inhibit viral replication [4].

Activating the IFN system is a complex, multistep process comprising (1) recognition of viral patterns (nucleic acids or proteins); (2) activation of adaptor kinases; (3) phosphorylation and nuclear translocation of transcriptional factors IRF3/IRF7; and (4) stimulation of IFN gene transcription. Secreted IFN molecules act as autocrine or paracrine mediators that stimulate IFN receptor complexes on the cell surface. The activation of IFN receptors stimulates the JAK/STAT signal transduction pathway and induces the expression of downstream ISGs [8,9]. Recombinant IFNs are approved for use as prophylactic or non-specific immunotherapy for a number of viral infections [10,11].

Notably, though quite effective in treating certain viral infections, therapeutic IFNs were developed several decades ago. Since then, immunology has made impressive progress, developing cytokine-based medications (e.g., recombinant IFNs [12]), next-generation chemical immunomodulators (agonists of Toll-like receptors [13,14] and antiviral sensors [15]) and biologics (therapeutic vaccines [15], chimeric antigen receptor (CAR) immune cells [16], retargeting and bispecific antibodies [17], etc.). However, in the never-ending evolutionary arms race between viruses and host immune responses, the latter is always at a disadvantage. Viruses have long been known to evade immunity, whilst the frequent mutations they acquire during replication in their host cells reduce the efficacy of antiviral therapies [18,19]. Due to the vast heterogeneity of viruses, the mechanisms of viral immune evasion are diverse and poorly described.

Modulating viral replication and modifying the immune response using novel molecular biology tools provide unprecedented means to study virus–host interactions and, possibly, build the foundation for new types of antivirals. In particular, adapting the bacterial defense system CRISPR–Cas for gene editing and beyond has already made these systems routine and very robust biological tools. CRISPR–Cas functions via binding of the Cas protein recruited to target DNA or RNA molecules by a short guiding RNA (sgRNA). Recognition of the target locus demands the presence of a short PAM sequence (two to seven nucleotides) immediately 3′ of the target site. Initially, pioneering studies of the recent Nobel Laureates J. Doudna and E. Charpentier demonstrated that the Cas9 protein can cleave target DNA by forming DNA double-strand breaks (DSB), so this technology can be used for programmed gene editing [20]. Less than 10 years later, the CRISPR toolkit has expanded dramatically and has been complemented with new CRISPR–Cas systems of different types, Cas proteins with modified PAM recognition (the PAMless Cas is possibly to be developed in the recent years), Cas proteins with increased or altered specificity, Cas nickases and related technologies (PrimeEditing, base editors), dead Cas proteins with additional functional domains (epigenetic modifiers, transcription activators/repressors), and other tools (reviewed in [21]). The invention of genome-wide CRISPR screens (CRISPRi, CRISPRa, CRISPRko) has allowed the examination of thousands of genes to identify their particular impact in human disease, including in infectious diseases [22]. Many CRISPR tools have been leveraged to develop novel antiviral approaches based on enhancing antiviral immune responses.

In this manuscript, we will review the main mechanisms of antiviral adaptive and innate immune responses and IFN systems, including recognition of viral patterns, activation of IFN secretion, and mechanisms by which viruses evade immunity. In the second part of the review, we will discuss the results and approaches used in recent studies to identify novel host factors, elucidate virus–host interactions, modulate antiviral immunity, and clarify the mechanisms of antiviral ISGs. Importantly, we also highlight recent developments in the use of CRISPR systems to enhance antiviral responses and their potential use as therapeutic agents in viral diseases.

## 2. The Role of Innate Immunity in Restricting Viral Replication

The innate immune system is the first line of defense against invading pathogens. Pathogen-associated molecular patterns (PAMP) can be sensed by pattern recognition receptors (PRR), resulting in the activation of signaling pathways that contribute to the elimination of these foreign agents [23]. One key player in these pathways is IFN. There are three families of IFN, with type I and III IFN serving as direct antivirals, and type II being the main regulator of antiviral innate and adaptive immune responses in different cell types [23]. Type I and III IFN activate the expression of ISGs, which have broad-spectrum antiviral activity [24].

### 2.1. Pattern Recognition Receptors (PRR)

Three main types of immune sensors for viral PAMPs (proteins and nucleic acids) have been discovered: Toll-like receptors (TLR), RIG-like receptors (RLR) (RIG-I, MDA5), cytosolic DNA sensors (DNA-PKcs, cGAS, AIM2 etc.), and nuclear DNA sensors (IFI16, hnRNPA2B1, cGAS etc.) [25,26,27,28]. TLR are localized on the cell surface (TLR1, TLR2, TLR4, TLR5, TLR6) and on the surface of endosomes (TLR3, TLR7, TLR8, TLR9). TLR2 and TLR4, anchored in the cell membrane, recognize viral proteins and induce downstream immune signaling. TLR3, TLR7/8, and TLR9 sense viral dsRNA (generated mostly as a by-product of viral replication), ssRNA, and CpG-rich DNA, correspondingly. Pathogen-derived RNA can also be detected by retinoic acid-inducible gene I (RIG-I)-like receptors (RIG-I and MDA-5) [29]. The main receptor that recognizes cytosolic DNA is cyclic GMP-AMP (cGAMP) synthase (cGAS) [30], but in various types of immune cells, foreign DNA can also be detected by the sensor-molecule “absent in melanoma 2” (AIM2) [28].

#### 2.1.1. Viral Sensing by TLR and RLR: Concise Overview

TLRs are transmembrane proteins containing three domains: ectodomain recognizing PAMP, transmembrane domain, and cytosolic Toll/IL-1 receptor (TIR) domain [31]. Once bound to their ligands, TLRs oligomerize via their TIR domains, recruiting adaptor proteins and initiating signal transduction.

**Figure 1:**
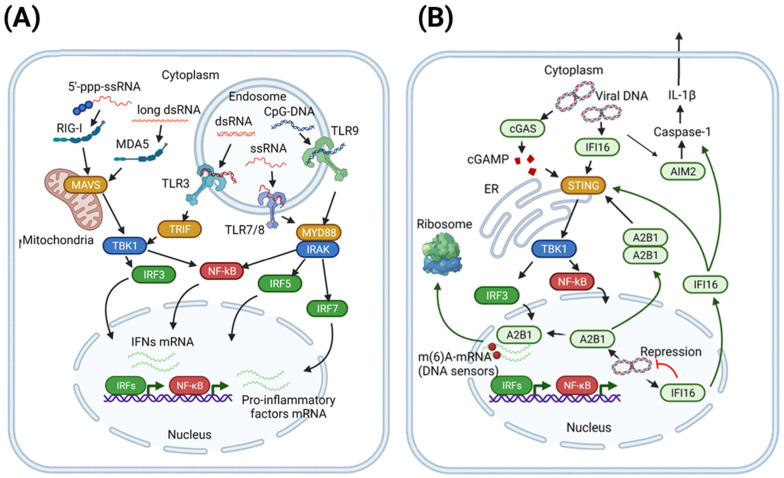
Sensing of foreign nucleic acids. (**A**) TLR- and RLR-mediated sensing of foreign nucleic acids. Different types of cytoplasmic foreign RNA are recognized by RIG-I or MDA5 sensors followed by activation of MAVS and downstream TBK1-IRF signaling. In endosomes, RNA and CpG-DNA activate TLRs that result in one of the two signaling pathways involving TRIF or MYD88-IRAK. Activation of IRFs induces the expression of interferons and mRNA of pro-inflammatory factors. (**B**) Cytoplasmic and nuclear sensors of foreign DNA. Cytoplasmic DNA can be sensed by a number of sensors, including IFI16, cGAS, and AIM2. The first two factors activate the STING pathway that ultimately induces TBK1/IRF and interferon secretion. Upon recognition of cytoplasmic DNA, AIM2 induces caspase-1-dependent maturation of pro-IL-1β (pro-inflammatory response). A2B1 (hnRNPA2B1) and IFI16, among other factors, can participate in the sensing of foreign DNA in the nuclei of cells. IFI16 interferes with foreign nuclear DNA by epigenetic silencing, whereas A2B1 recognizes foreign DNA as well as activates and enhances innate antiviral responses. This picture was created in BioRender. Abbreviation: A2B1—hnRNPA2B1; m6a—methyl-6-adenine RNA; ER—endoplasmic reticulum.

Depending on the adaptor proteins recruited to activated TLRs, downstream signaling occurs via “myeloid differentiation primary response 88” (MyD88)-dependent or TIR domain-containing adaptor-inducing interferon-β (TRIF)-dependent pathways (Figure 1A). Most TLRs recruit MyD88 adaptor, but TLR3 recruits TRIF [32]. MyD88 forms a signaling complex with kinases IRAK4 and IRAK1/2 [33], leading to IRAK4 trans-autophosphorylation followed by the activation of IRAK1/2. In turn, this complex phosphorylates and activates NF-κB, IRF5, and IRF7 [34]. TRIF interacts with TRAF, resulting either in TBK-1 activation with subsequent IRF-3 phosphorylation or in IkB degradation and the release and activation of NF-κB transcription factors. The main effect of NF-κB activation is the secretion of pro-inflammatory cytokines and activation of pro-IL-1β. IRF3 and IRF7 primarily result in induction of the IFN response, while IRF5 acts both as a pro-inflammatory factor and an IFN inducer.

Signaling through RIG-I and MDA5 receptors, which recognize cytosolic RNA, is essential for eliciting IFNα/β responses and, ultimately, clearing or inhibiting the incoming virus (Figure 1A). RIG-I and MDA5 signal through “mitochondrial antiviral-signaling protein” (MAVS; also known as IPS-1, VISA, and CARDIF) [29,35]. This increases the production of type I IFNs and pro-inflammatory cytokines. IFNs in turn induce ISG expression and suppress viral infection. In addition, the overactivation of mitochondrial MAVS adaptor protein leads to IFN-independent and Caspase-9-dependent activation of apoptosis in infected cells. The pro-apoptotic activity of MAVS can often be abrogated by different viral proteins as a mechanism of viral immune evasion [36].

RIG-I recognizes mainly blunt-end short RNAs with 5-triphospate [37], and MDA5 senses long viral dsRNA. RNA binding changes the conformation of RIG-I and MDA5: RIG-I forms tetramers, whereas MDA-5 oligomerizes. RIG-I and MDA-5 activation depends on the binding of their CARD domain to K63 polyubiquitin chains [38]. TRIM25 and RIPLET ubiquitin ligases synthesize polyubiquitin chains, promoting RIG-I signaling. Activated RIG-I and MDA-5 recruit MAVS for further signaling. Upon activation, MAVS aggregates on the mitochondrial surface [39]. When small aggregates are assembled, they can recruit other MAVS molecules to form large aggregates. MAVS activates signaling through IKK and TBK1 kinases, leading to NF-κB and IRF3 activation. Ubiquitin ligases TRAF2, TRAF5, and TRAF6 also can be recruited to MAVS and activate downstream signaling [40]. It is well established that RIG-I triggers the innate immune response during infection by orthomyxoviruses, paramyxoviruses, rhabdoviruses, and other viruses [41]. RIG-I is the major PRR that initiates the host antiviral response against hepatitis C virus (HCV) via recognition of poly-U/UC motifs in HCV RNA [42]. RIG-I was shown to be important both for sensing hepatitis B virus (HBV) RNA (more specifically, the 5′-ε region of HBV pre-genomic RNA, the major form of HBV RNA), but it is also a direct antiviral factor that impairs interaction between HBV polymerase and pre-genomic RNA [43].

The induction of miR146a by HBV was shown to attenuate antiviral innate immune responses by targeting RIG-I and RIG-I enhancer. Impairment of RIG-I and RIG-I enhancer signaling by HBV may be one of the major mechanisms responsible for the evasion of host immunity by HBV [44]. Similarly, activation of RIG-I was shown to inhibit human immunodeficiency virus (HIV) replication in macrophages by inducing the expression of critical ISGs such as APOBECs, tetherin, and CC chemokines [45]. However, HIV also developed a protease that counteracts RIG-I signaling: the expression of HIV protease promotes the loss of cytoplasmic RIG-I by sequestering it in lysosomes [46].

#### 2.1.2. Foreign DNA Recognition by Cytosolic and Nuclear DNA Sensors

The cytosolic sensor of DNA is cyclic GMP-AMP synthase (cGAS), which contains two major DNA-binding domains and a nucleotidyltransferase domain [30]. cGAS binds DNA independently of the nucleotide sequence [30], instead targeting its sugar-phosphate backbone or recognizing Y-shaped structures of ssDNAs [47,48]. Upon binding to DNA in the cytosol, cGAS synthesizes a second messenger cGAMP from ATP and GTP [30], which activates the adaptor “stimulator of interferon genes” (STING). STING protein contains four transmembrane domains and is localized on the endoplasmic reticulum. STING itself cannot bind DNA, but it undergoes a conformational change upon cGAS binding and translocates to the nuclear compartment for TBK1 and IKK complex activation [49]. That leads to the activation of transcription factors IRF3 and NF-κB, resulting in the expression of type I IFN and pro-inflammatory cytokines [50].

Cytosolic DNA can also initiate inflammasome formation, which is a platform for pro-inflammatory cytokine maturation, via recognition by AIM2-like receptors (ALRs) [28,51]. Nucleic acid recognition leads to AIM2 dimerization and further interaction with apoptosis-associated speck-like protein containing a CARD (ASC) [52] followed by the activation of caspases [53].

Although initially enigmatic, in recent years, a plethora of potential nuclear sensors of foreign DNA were identified, including cGAS, IFI16, hnRNPA2B1, DA/ZBP1, TLR7/9, ZCCHC3, RNA Pol III, etc. [54]. Among them, for cGAS (recognition of DNA double-strand breaks), IFI16 (functions as a transcriptional repressor of foreign DNA), and hnRNPA2B1 (activates and amplifies antiviral response), DNA-sensing activity was directly determined (Figure 1B). More detailed information about the functioning of nuclear DNA sensors can be found in recent reviews [54].

### 2.2. Restriction of Viral Replication by Interferon-Stimulated Genes (ISGs)

**Figure 2:**
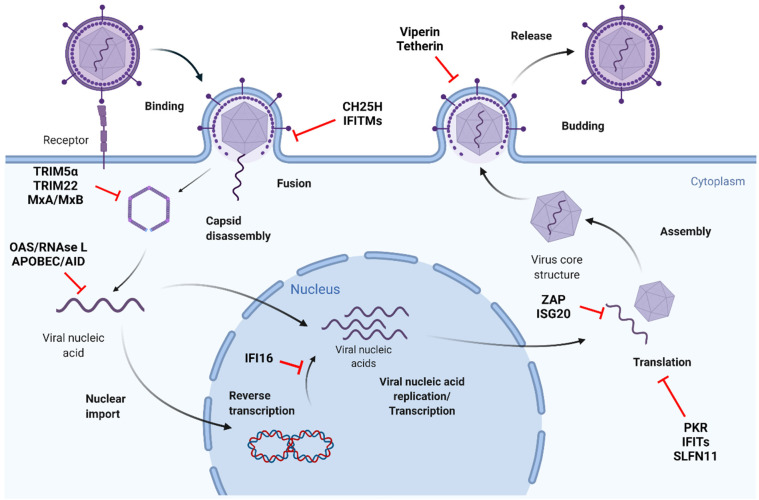
Restriction of viral life cycle by different ISGs for viruses with nuclear replication. ISGs with antiviral activity are shown for different steps of viral replication, including binding and viral entry, capsid disassembly, nuclear import, reverse transcription, viral nucleic acid replication/transcription, nuclear export, translation, capsid assembly, budding and release of viral particles. This picture was created in BioRender.

Upon activation, PRRs initiate a signaling cascade that leads to IFN production. In turn, IFNs activate the JAK–STAT signaling pathway, resulting in the subsequent expression of numerous ISGs with broad antiviral activity. ISGs can restrict virtually every step of the viral life cycle (Figure 2).

#### 2.2.1. Restriction of Viral Entry

Viral entry is commonly inhibited by such ISGs as CH25H, which converts cholesterol to 25-hydrocholesterol (25HC). 25HC changes the composition of the cell membrane, thus blocking membrane fusion between the virus and cell [55]. CH25H has broad antiviral activity and reduces infection by vesicular stomatitis virus (VSV), herpes simplex virus (HSV), HIV, HCV, Ebola virus (EBOV), Nipah virus, Zika virus (ZIKV), and other viruses [55,56,57]. Another factor that inhibits viral endocytosis is human nuclear receptor coactivator 7 (NCOA7), which binds vacuolar H^+^-ATPase, resulting in the degradation of viral particles [58]. Interferon-induced transmembrane protein (IFITM) family proteins have also been shown to block viral infection at the stage of viral fusion and cytosolic entry. IFITM proteins have broad antiviral tropism, including influenza A virus (IAV), dengue virus (DENV), West Nile virus (WNV), EBOV, Marburg virus, SARS-CoV, SARS-CoV-2, rhabdovirus, bunyavirus, HCV, HIV, and others [59,60,61,62,63,64,65,66,67,68]. Most recently, screening of the ISG library revealed that lymphocyte antigen 6 complex, locus E (LY6) potently restricts infection by multiple coronaviruses, including SARS-CoV-2 [69].

#### 2.2.2. Restriction of Protein Translation

Many ISGs impair viral protein translation. Protein kinase R (PKR) is widely known to inhibit the production of viral proteins [70]. The family of IFIT proteins inhibits the translation of viral proteins by different mechanisms, for example, by blocking the initiation of translation [71]. ISG15 can co-translationally conjugate with viral proteins [72]. Recently discovered ISG Schlafen 11 (SLFN11) inhibits rare tRNA codons, which are sometimes used by viruses [73]. Another mechanism is employed by an ISG Shiftless, which inhibits the ribosomal frameshifting used by HIV to regulate the ratio of its proteins [74].

#### 2.2.3. Restriction of Viral Replication

Many ISGs can restrict viral replication. Viperin blocks DNA and RNA viruses by binding viral proteins and thus preventing their replication [75]. The process of reverse transcription can be targeted by apolipoprotein B mRNA-editing enzyme catalytic polypeptide-like (APOBEC3G), which induces mutations in the viral genome [76]. IFI6 was shown to disrupt replication flavivirus organelles [77], while RBBP6 protein impairs transcription of EBOV [78].

Several ISGs can destabilize and destroy viral RNAs. Oligoadenylate synthetases (OAS) catalyze the formation of 2′–5′-linked oligoadenylates that activate cellular RNase L, resulting in the degradation of viral RNA genomes [79]. Endonuclease ZAP can inhibit viral replication by preventing the accumulation of mRNA in the cytoplasm [80]. APOBEC3A, APOBEC3B [81] and AID [82] have been shown to directly deaminate and destroy HBV nuclear depo, covalently closed circular DNA, thus paving the ways for developing novel anti-HBV therapeutics. ISG20 can interfere with viral replication by several mechanisms. ISG20 can impair mRNA synthesis and protein translation of RNA viruses [83,84]. Potentially, ISG20 can also contribute to the restriction of HBV replication and degradation of HBV cccDNA by APOBEC3A [85].

#### 2.2.4. Induction of Inflammatory Response

TLR activation can result in the maturation and migration of immune cells (dendritic cells), enhanced phagocytosis and generation of reactive oxygen species (macrophages and neutrophils), overproduction of co-stimulatory molecules (e.g., B cells), and other immune responses [86]. TLR activation must be tightly regulated for adequate innate immune response to pathogen DNA, and dysregulated TLR signaling is associated with chronic inflammatory conditions and, in some cases, septic shock [87,88,89].

Overall, the effects of ISGs and their mechanisms of action are diverse, with some playing a major role in the restriction of viral agents, while some being dispensable (or with an incremental effect) for the antiviral response. It is noteworthy that the effects of ISG interaction networks in some cases may have cumulative effect that, in certain infections, could be more important for viral restriction than effects of individual ISGs.

### 2.3. Host Factors Targeted by Viruses for Immune Evasion

**Figure 3:**
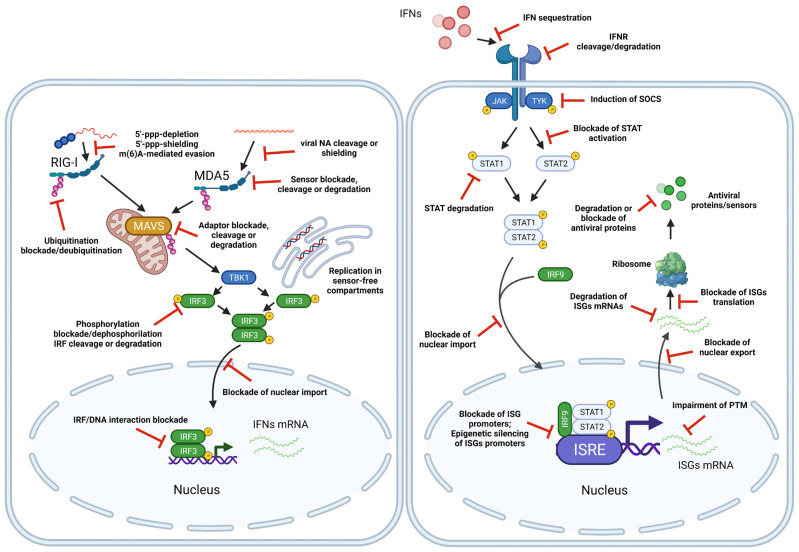
Mechanisms of viral immune evasion. (**A**) Evasion of immune recognition. (**B**) Blockade of interferon signaling. This picture was created in BioRender. Abbreviations: NA—nucleic acids; ISG—interferon-stimulated genes; IFNs—interferons; PTM—post-transcriptional modifications; SOCS—suppressor of cytokine signaling.

The limited capacity of host cells to sense pathogen nucleic acids is explained by the existence of elaborate immune evasion mechanisms utilized by viruses, which have evolved a myriad mechanism allowing them to avoid recognition by immune sensors; some of these mechanisms are summarized in Table 1 and depicted in Figure 3.

Many groups of viruses can inhibit PRRs themselves. For example, vaccinia virus (VACV) was reported to block TLR9 signaling by binding viral protein A46R to the MyD88 adaptor [90]. Human cytomegalovirus (HCMV) disrupts TLR signaling pathways with its protein US7, which promotes the ubiquitination of TLR3 [91]. Kaposi’s sarcoma-associated herpesvirus (KSHV) and HBV can block TLR2, TLR4, and TLR9 expression, reducing the levels of pro-inflammatory cytokines [92,93].

**Table 1:** Host factors targeted by viruses and mechanisms of immune evasion.

Target	Virus	Viral Protein	Mechanism
RIG-I	Influenza virus	NS1	Direct interaction, TRIM and RIPLET binding
	Coxsackievirus B3	2Apro	Cleavage
	Epstein-Barr virus	LMP1	Proteasomal degradation
	SARS-CoV	Nucleocapsid protein	Binding TRIM25
	Respiratory syncytial virus	NS1	Binding TRIM25
	Human papilloma virus	E6	Proteasomal degradation of TRIM25
	WNV	NS1	Proteasomal degradation of TRIM25
	HCV	NS3-4A	Cleavage of RIPLET
	DENV/WNV	NS3	Binding 14-3-3ε
TLR9	Vaccinia virus	A46R	MyD88 adaptor binding
TLR3	Human cytomegalovirus (HCMV)	US7	Resultant ubiquitination of TLR3
	HIV	No data	Inhibits phosphorylation of IRF3, IRF7, STAT1, STAT3
MDA5	Measles virus	V	Prevention of MDA5 dephosphorylation
	SARS-CoV-2	NSP8	Impairment of K63-linked polyubiquitination
	DENV/WNV	NS3	Binding 14-3-3ε
IFI16	HSV1	ICP0/Ul41	Ubiquitinoylation/inhibition of expression
	CMV	pUL83	Direct interaction
cGAS	HSV1	Ul41/Ul37/Vp22	Inhibition of expression or enzymatic activity
	HCMV	UL31/pp65	Direct interaction/enzymatic activity inhibition
	Kaposi’s sarcoma-associated herpesvirus (KSHV)	ORF52/LANA	Direct interaction/enzymatic activity inhibition
	ZIKV	NS1	Stabilization of caspase1
TRIF	HCV	NS3-4A	Cleavage
MAVS	DENV	NS2B3	Binding of MFN1 and MFN2 proteins
	Rhinoviruses	2A and 3C	Cleavage
STING	Adenovirus	E1A	Binding of STING
	HPV18	E7	Binding of STING
	KSHV	vIRF1	Prevention of STING interaction with downstream factors
	HBV	Pol	Prevention of STING polyubiquitylation
	HCV	NS4B	Inhibition of downstream signaling
	HIV	Vpx	Antagonizes cGAS/STING-triggered NF-κB signaling [94]
	DENV	NS2B3	Inhibition of downstream signaling
	Yellow fever virus	NS4B	Inhibition of downstream signaling
IKKε	Lassa fever virus	Nucleoprotein	Inhibition of autocatalytic activity
	EBOV	Vp53	Direct binding
TBK1	HIV	Vpr and Vif	Direct binding
	EBOV	Vp53	Direct binding
	Human herpesvirus 8	vIRF1	Interaction with CBP/p300
	HSV1	ICP34.5	Binding of TBK1
	KSHV	ORF45	Alternative substrate for TBK1
IRF7	Enterovirus 68	3Cpro	Cleavage
STAT2	DENV	NS5	Ubiquitination
IRF3	SARS-CoV	ORF3b, ORF6 and N	IRF-3 inhibition

Other viruses have been shown to disrupt signaling by MDA5 and RIG-I, which are two crucial RNA sensors responsible for viral recognition in the cytoplasm. Enteroviruses (e.g., coxsackievirus B3) encode 2A and 3C proteases that cleave MDA5 and RIG-I [95]. Negative-sense, single-strand RNA measles virus (MV) can inhibit MDA5 activation by its V protein, which binds to phosphatases PP1α and PP1γ and prevents the dephosphorylation of MDA5 during infection [96]. Similarly, Epstein–Barr virus (EBV) encodes «latent membrane protein» (LMP1) that mediates RIG-I proteasomal degradation [97], whereas influenza virus NS1 protein can interact with RIG-I and impair its function [98]. One of the mechanisms whereby SARS-CoV-2 inhibits innate immunity is by suppressing of MDA-5 by nonstructural protein NSP8 [99].

Some viruses do not interact with MDA5 or RIG-I directly but rather counteract the activity of factors responsible for RIG-I activation, such as TRIM25 and RIPLET (mediate RIG-I ubiquitinoylation) and 14-3-3ε. Influenza virus protein NS1 binds both factors and decreases the production of IFNs [100], while SARS-CoV nucleocapsid protein and respiratory syncytial virus NS1 protein target TRIM25 and prevent the activation of RIG-I [101,102]. TRIM25 can also be targeted to proteasomal degradation by HPV’s E6 protein and WNV’s NS1 [103,104]. HCV proteases NS3-4A can cleave RIPLET and thus impair RIG-I activation [105]. The third factor, 14-3-3ε, can be bound by NS3 proteases of DENV and WNV that block subsequent immune response activation [106].

Viral DNA can be recognized and sensed not only by cytoplasmic (cGAS/STING), but also by nuclear DNA sensors, including IFI16, which can sense DNA viruses replicating in the nucleus [107]. Many viruses elaborated mechanisms to dampen IFI16 activity. For instance, immediate early protein ICP0 of HSV-1 ubiquitinylates IFI16, promoting its degradation [108,109]. The matrix protein pUL83 of HCMV blocks IFI16 signaling via direct interaction [110], while HCMV UL41 protein inhibits IFI16 and cGAS expression at the mRNA level [111,112].

ORF52 of KSHV and NS1 protein of ZIKV were likewise shown to inhibit cGas protein [113] and promote its degradation [114]. Similarly, Vp21 and the matrix protein UL37 of HSV1 inhibit cGAS [115,116]. HCMV UL31 interacts with cGAS, and its matrix protein pp65 binds cGAS to prevent its interaction with STING [117,118]. LANA protein of KSHV can also inhibit cGAS by direct interaction [119].

Viruses also can act on downstream factors of immune signaling pathways. In particular, NS proteases of HCV block several transcription factors involved in regulating innate immune responses. NS3-4A inhibits transcriptional factor IRF3 by binding MAVS [120] and cleaves the TRIF protein [121]. The NS2B3 protease of DENV binds and blocks MFN1 and MFN2 proteins, which are important regulators of MAVS [122]. MAVS is also targeted for cleavage and degradation by 2A and 3C proteases of rhinoviruses [123].

As already mentioned, STING is a crucial component of the cGAS–STING signaling axis that is responsible for detecting viral nucleic acids and deploying antiviral defense responses. Many DNA viruses interfere with the function of STING by cleaving it or impairing its ubiquitination (an important post-translational modification required for its function and for the regulation of innate immune responses). For example, adenoviral E1A protein and HPV18 E7 protein are known STING pathway inhibitors [124]. vIRF1 protein of KSHV prevents STING pathway activation by interacting with TBK1 and IRF3, which is the target of TBK1 and an important transcriptional factor required for IFN induction [125]. HBV polymerase prevents STING polyubiquitylation, reducing STING function [126] and impairing IFN-β induction. Surprisingly, RNA viruses also can affect the DNA-sensing STING pathway. This is significant, because many RNA viruses not directly detected by cytoplasmic or nuclear DNA sensors can induce the release of nuclear or mitochondrial DNA into the cytoplasm, followed by activation of the cGAS–STING axis and pro-inflammatory and antiviral innate responses. For instance, HCV NS4B protein, DENV NS2B3 protease, and yellow fever virus (YFV) NS4B protein all block STING downstream signaling and impair the IFN response [127].

IKKε and TBK1 kinases are signal transducers in MAVS and STING signaling pathways. IKKε is frequently targeted by viral proteins, including Lassa fever virus nucleoprotein [128], whereas TBK1 is inhibited by Vpr and Vif protein of HIV [129]. Both IKKε and TBK1 are blocked by EBOV Vp35 protein [130]. Herpesvirus ICP34.5 protein can bind TBK1, resulting in decreased IFN type I expression [131].

HHV-8 protein vIRF1 prevents the interaction of STING and TBK1, thereby inhibiting STING, TBK1, and IRF3 activation. This protein can also interact with transcriptional activator CBP/p300, impairing CBP/p300 and IRF3 association and reducing the efficacy of transcriptional activation from IRF3-dependent promoters [132]. A different strategy for TBK1 inactivation is used by KSHV protein ORF45, which competes with IRF3 as a TBK1 substrate, stopping innate immune signaling at the TBK1–IRF3 binding step [133].

Viruses can inhibit transcriptional factors that mediate IFN induction and ISG activation. Enterovirus 68 (EV-D68) 3Cpro cleaves IRF7 during infection [134]. DENV NS5 protein leads to the ubiquitination and degradation of STAT2 [135,136], which is a factor of signal transduction from receptor to IFN-I/III. NS5 can also inhibit IFN signaling by cleaving STAT2 [137]. SARS-CoV proteins ORF3b, ORF6, and N have been shown to antagonize the IFN pathway mostly by inhibiting IRF-3 protein [138]. ZIKV has been demonstrated to suppress IFNβ in vitro [139].

Chikungunya virus (CHIKV) effectively blocks the translation of ISG mRNAs, preventing the antiviral immune response [140]. One of the important mediators of this process may be the viral nsP2 protein, which could be involved in inhibiting STAT signaling [141]. Several studies reported that cellular nucleoporins are destroyed and mis-localized and that nucleo-cytoplasmic trafficking pathways are disrupted in cells infected with rhino- and polioviruses. The 2A protease of rhinoviruses (poliovirus or other enteroviruses) can cleave translation initiation factor elF4G, resulting in the translational shutdown of cellular mRNAs [142]. In addition, 2A protease cleaves nuclear pore proteins Nup62 and Nup98, while 3C protease cleaves Nup153 [135,143]. These cleavage events alter the efficacy of the host immune response signaling and promote immune evasion.

HIV Vif protein is widely known to block antiviral host responses by mediating the proteasomal degradation of APOBEC enzymes [144]. Tetherin, a host transmembrane IFN-induced protein, blocks the detachment and release of enveloped viruses. HIV can evade the antiviral activity of tetherin by expressing the Vpu protein, which binds tetherin and inhibits its activity [145].

To conclude, viruses develop elaborate mechanisms to evade or become resistant to innate antiviral response. Identifying factors able to abolish viral replication and contribute to viral clearance is important for developing novel therapeutics.

## 3. Emerging CRISPR–Cas Tools

Since their adaptation for genome editing, CRISPR–Cas systems have become one of the most widespread tools in molecular biology. The most commonly used CRISPR–Cas system is CRISPR–Cas type II, in which the Cas9 protein introduces a DSB in the target DNA site via nucleolytic activity of RuvC and HNH domains [20]. Resulting DSBs can be repaired predominantly by homologous recombination (HR) or non-homologous end joining (NHEJ), or occasionally by alternative pathways [146,147,148]. NHEJ is an error-prone DSB repair mechanism that results in out-of-frame mutations at the DSB site and, as a consequence, leads to gene inactivation. Alternatively, in the presence of a homologous DNA template, DSBs can be repaired by HR, which preserves the integrity of the genome [149]. The main disadvantages of CRISPR–Cas-mediated cleavage include (a) potential genotoxicity [150,151], the formation of large (up to several kb) deletions at the target site and extensive chromosome aberrations, including chromothripsis [152,153]; (b) low efficacy of DNA integration by the HR mechanism [154]; (c) intracellular responses to CRISPR–Cas components [155]; and (d) potential involvement of repair pathways other than NHEJ or HR that may yield unpredictable on-target mutations [146,156].

Inactivating Cas nucleolytic activity by single-point mutations in RuvC and HNH nuclease domains generates a nuclease-null or “dead” Cas (dCas) system [157]. The dCas protein retains its ability to bind to the target site but cannot generate DSBs. Developing chimeric dCas-X systems, in which X is any functional domain, endowed CRISPR–Cas with numerous additional functional modalities and enabled unprecedented manipulation of both the genome and the epigenome [21].

### 3.1. Modulation of Gene Transcription by CRISPR

CRISPR-activation (CRISPRa) or interference (CRISPRi) systems are based on fusing transcriptional activators/repressors to dCas [157]. The system is recruited to the regulatory regions of target genes (promoters or enhancers) to transactivate or suppress gene transcription. The major advantages of the CRISPRa approach over overexpressing cDNA are the precise, tunable control of target gene expression and ability to overexpress selected or all isoforms of a gene.

First-generation CRISPRa systems include dCas9–(VP16)n [158], dCas9–p65–HSF1 [159], and dCas9–p300 [160], and they are characterized by relatively low activation efficiency. Robust activation of target gene transcription is feasible when using several sgRNAs targeting an extended genomic region. Historically, one of the first activators was based on herpesvirus protein VP16 domains, which transactivate gene expression by recruiting pre-initiation complex factors to the dCas9–VP16-bound regions. The signal produced by VP16-based CRISPRa systems can be further amplified by fusing multiple VP16 domains (3–10 units) [158,161]. The transcription factor p65-HSF1 is another example of directly activated gene transcription. Another CRISPRa system takes advantage of p300 acetyltransferase, which modifies the epigenetic state of the promoter and enhancer regions by directly acetylating histone H3 at lysine K27 [160]. This acetylation induces gene transcription and may also result in the recruitment of additional transcription factors to the region of interest.

Second-generation CRISPRa systems include Scaffold [162], VPR [163], SunTag variants [164], SAM [165], and more recently developed dCas9–CBP [166] and SPH [167]. Compared to first-generation systems, these systems are characterized by improved on-target gene activation, and they allow the multiple overexpression of genes with a single sgRNA. The key property of second-generation CRISPRa systems is that they mobilize several activation domains. They rely on different strategies, such as dCas9 fused to various activation domains. The principle of second-generation activators is the recruitment of several activation domains to target sites. The VPR activator consists of VP64 herpes virus protein, p65 transcriptional factor, and Rta (transcription factor of Epstein–Barr virus). In the SunTag system, GCN4 peptide arrays are fused to dCas9. In the same cell, activation domains (VP64, p300, p65-HSF1, or others) fused to GCN4-scFv are produced. GCN4-scFv is a single-chain antibody fragment with a high affinity for GCN4 arrays. As a result, up to 10 copies of hybrid Activator-scFv domains are recruited to the dCas9 binding site to activate transcription. The scaffold uses unmodified dCas9 protein and sgRNA modified with two special RNA aptamers (e.g., MS2 aptamers). Additionally, activators fused with the aptamer-specific protein are produced (MCP protein in case of MS2). Four activator–MCP domains are recruited to sgRNA aptamers in the dCas9 binding site for transcriptional induction. In SAM, dCas9 protein is modified with VP64 activator and attracts four p65–HSF1 activation domains to aptamer-modified sgRNA for pronounced transcriptional activation (reviewed in [21]).

The most widely used CRISPRi system is dCas9-KRAB [168]. The KRAB inhibitory domain attracts epigenetic enzymes that mediate the deposition of inactive chromatin marks (H3K9Me3 and H3K27Me3) to the target site (gene promoters or enhances), subsequently repressing transcription. Alternatively, the EZH2 inhibitory domain may be used [169].

### 3.2. CRISPR-Based DNA- and RNA-Editing Tools

Base editors (BE) are molecular tools that introduce pinpoint nucleotide replacements into the target DNA site. Functional domains of BE are cytidine and adenosine deaminases. Instead of dCas proteins, nCas9 with only one inactivated nuclease domain is used in the latest BEs. After binding to the target site, deaminases catalyze the editing of nucleotides in a narrow nucleotide window of one DNA strand and nicking of the complementary strand. Cytidine BEs catalyze cytidine deamination and generation on uridine (U). After the nicked strand and deaminated nucleotides are repaired, U is changed to a thymine (T) residue. Adenine BEs generate inosine (I) instead of adenine, resulting in guanine (G) after repair. Thus, cytosine BEs mediate C→T/G→A editing and adenine BEs mediate A→G/T→C editing. Base editing results in very infrequent insertion/deletion (indel) mutations and thus a safer analog of the gene editing systems. CRISPR-STOP and iSTOP technologies use cytidine BE to create stop codons in early exons of genes, knocking out gene expression without introducing DSB. More extensive descriptions and characterization of different CRISPR–Cas tools can be found in other reviews [170].

In the recent years, several RNA editing tools based on RNA-specific CRISPR–Cas13 were developed. Cas13 proteins are specific to RNA, interacting with target moieties by CRISPR RNA (crRNA), and they exhibit ssRNA-specific nuclease activity. Cas13-based approaches have already been used to target viral RNA and inhibit the replication of ssRNA viruses, including LCMV, IAV, VSV, SARS-CoV-2, etc. [171,172]. Nucleolytically null dead Cas13 proteins (dCas13) were generated and used as platforms to develop RNA base editors, such as REPAIR (utilizes an evolved variant of ADAR2 adenine deaminase to deaminate nucleotides). dCas13 serves as a vehicle to recruit ADAR2 to the crRNA:target RNA duplex. Next, ADAR2 mediates A→I conversion in target RNA [173].

Evolved ADAR2 protein exhibits dual A→I and C→U deaminase activity (RESCUE system) [174]. By replacing ADAR2 deaminase domain with APOBEC3A deaminase, it was possible to generate a C→U RNA-specific editase (CURE system) [175]. Additionally, truncated variants of Cas13 RNA editing tools (<1000 aa) that can be easily packaged into common AAV were engineered [176]. These advancements have become a prominent milestone on the path to create CRISPR-based therapeutics.

Differences in the properties and applications of DNA and RNA base editors were reviewed elsewhere [21]. A short summary about CRISPRa/CRISPRi and base editing systems is listed in Table 2.

Different variants of CRISPR–Cas systems are widely used in basic and translational research. Current directions of CRISPR–Cas applications in antiviral immunity can be divided into three areas: screening for potent antiviral genes, basic research into antiviral immunity, and development of antiviral approaches, which we review below.

## 4. CRISPR–Cas Systems for Modulating Antiviral Responses

### 4.1. Screens to Identify Antiviral Genes

**Figure 4:**
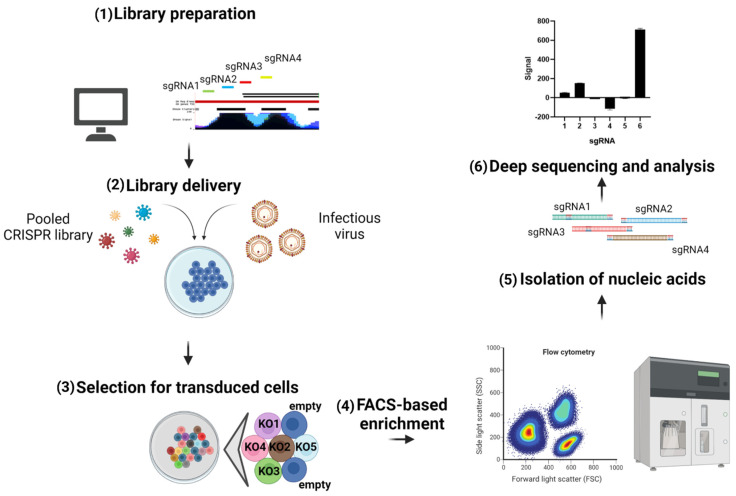
General pipeline of pooled CRISPR screens. (**1**) In silico design of sgRNA targeting regulatory elements (promoters and/or enhancers) of target genes (or in genome-wide format). In modern pooled libraries, four to 10 different sgRNAs are designed to target a single regulatory element, which constitutes up to 200,000 sgRNAs in a single genome-wide library. (**2**) Lentiviral delivery of CRISPR pooled library into cells expressing appropriate Cas protein (Cas9 for knockout screens and dCas9 for activation of interference screens) into infected cells at low MOI (to ensure delivery of a single sgRNA into a single cell). (**3**) Selection of transduced cells (negative or positive). Note that knockdown of genes (KO) is relevant for nucleolytic Cas systems and base editors, whereas CRISPRa and CRISPRi systems cause the overexpression or suppression of gene transcription, correspondingly. (**4**) Enrichment of cells. (**5**) Isolation of nucleic acids from the bulk of enriched cells. (**6**) Deep sequencing and identification of hits by determining sgRNAs representation in the material. This picture was created in BioRender.

The major advantage of CRISPR screens is that they are high throughput and enable full-genome analysis of overexpressed or silenced genes (general pipeline of the pooled CRISPR screen is highlighted in Figure 4). In this way, CRISPR-mediated screening has great potential for identifying host factors with potent activity against different viruses. In addition to the genome-wide format, a plethora of libraries for studying specific sets of genes has been created, including pooled sgRNA libraries for assaying ISGs. Potential antiviral genes can be identified in loss-of-function (CRISPR knockout with Cas9 nucleases) or gain-of-function (CRISPRa) formats [184]. For some studies, loss-of function screens require treating experimental cell lines with certain cytokines (e.g., type I IFN) to obviate differences in the functional effects and gene expression, followed by knocking out specific genes and comparing to control samples [77]. The main studies that used CRISPR screens to identify host factors responsible for viral replication and identifying novel antiviral genes are listed in Table 3.

Zhu et al. (2018) performed CRISPR-based screening to identify factors that mediate the silencing of unintegrated retroviral DNA. Integrase-deficient, GFP-tagged Moloney leukemia virus (MLV) was used as the in vitro model. The group identified NP220, HUSH complex, and SETDB1 histone methyltransferase as the main factors required for silencing of unintegrated retroviral DNA. These factors were shown to recruit epigenetic silencers that remove active chromatin markers from MLV DNA and introduce heterochromatin markers, including H3 histone deacetylation (mediated by HDACs) and H3K9Me3 deposition (mediated by SETDB1) [185].

Another genome-wide CRISPR knockout screening to identify viral restriction factors was conducted by Richardson et al. (2018) using a model of Venus-GFP-expressing YFV. In this screen, hits included IFN signaling pathway factors (IFNAR1, IFNAR2, IRF9, and others), mRNA processing factors (ECD, MFAP1, etc.), and ISG effector gene IFI6. Experiments in cells with knocked out IFI6 confirmed the inhibitory effect of this gene on other flaviviruses, including WNV, DENV, and ZIKV. Mechanistically, IFI6 prevents the formation of endoplasmic reticulum single-membrane invaginations that are exploited by flaviviruses for replication, thus suppressing the viral life cycle. Notably, IFI6 does not inhibit HCV flavivirus or coronaviruses, which form ER-derived double-membrane vesicles during replication. Another screening hit, HSPA5, encodes BiP, which is a heat shock protein 70 chaperone that assists in protein folding and surveillance of misfolded proteins. BiP facilitates proper folding and/or localization of IFI6 at the membrane of the endoplasmic reticulum, thus supporting IFI6 antiviral activity against flaviviruses. Knocking out BiP resulted in IFI6 degradation [77].

CRISPR knockout screen, reported by Chia et al. (2020) using IAV after IFNβ treatment, revealed RTF2 as a potent factor with anti-IAV activity. Hit validation experiments demonstrated that RTF2 antiviral activity depends on nuclear localization and IFN. Although in this study, RTF2 expression did not increase after IFN treatment, IAV infection led to lower RTF2 levels, which may indicate a viral immune evasion mechanism. The antiviral effect of RTF2 is mediated by the induction of ISGs after IFN treatment, and loss of RTF2 leads to reduced levels of phosphorylated STAT and diminished ISG activation [186].

A CRISPR-mediated screen of anti-HIV ISGs was performed by OhAinle et at. (2018) using modified lentiviral vectors to deliver sgRNAs [187]. Lentivectors packaged sgRNA-encoding sequences into budding HIV particles in trans, resulting in the secretion of HIV–CRISPR particles into cultured media. Deep sequencing analysis of sgRNAs in secreted HIV–CRISPR virions was compared to the original sgRNA library; the disappearance of a certain sgRNA from the resultant HIV–CRISPR virions suggested that the gene targeted by this sgRNA exhibited anti-HIV activity. This approach can be used to identify viral restriction factors and host dependency factors. This study also demonstrated that some host factors (MxB, TRIM5α, UBE2L6, IFITM1) restrict the replication of multiple HIV isolates, while others are active only against specific HIV strains. These differences could be explained by immune evasion mechanisms utilized by certain HIV strains during chronic infection [187].

In another CRISPR screen targeting 1906 human ISGs with eight sgRNAs per gene, Roesch et al. (2018) identified IFITM factors as potent inhibitors of lentiviral particle delivery [188]. IFITM1/3 displayed an evident antiviral effect in a model of VSV-g pseudotyped viral-like particles encoding the HIV Vpx gene; Vpx-mediated SAMHD1 degradation was used as a readout. Flow cytometry and the sorting of cells with the lowest SAMHD1 expression followed by deep sequencing of sgRNAs identified IFITM1/3 as hits. VSVg-pseudotyped particles were significantly more sensitive to IFITM restriction than wild-type HIV with its natural envelope. The described screening approach can be used to identify restriction factors specific for different viral envelopes [188].

**Table 3:** Major screening studies of host antiviral restriction factors.

CRISPR Screen Type	Model Virus	Most Potent Factors Identified	Ref.
CRISPR KO	Integrase-deficient MLV	NP220, HUSH complex	[185]
CRISPR KO	YFV and other flaviviruses	IFI6, HSPA5	[77]
CRISPR KO	IAV	IFNAR2, TYK2, JAK1, IFNAR1, IRF9, RTF2	[189]
CRISPR KO (ISGs only)	HIV	MxB, TRIM5α, IFITM1, tetherin, SAMD9L, UBE2L6	[187]
CRISPR KO (ISGs only)	VSVg-pseudotyped viral-like particles	IFITM1, IFITM3	[188]
CRISPRa	MNoV	TRIM7, GBP2, MX1	[190]
CRISPRa	ZIKV	IFI6, IFNL2, ISG20, HELZ2	[191]

Abbreviations: CRISPR KO, CRISPR knockout; CRISPRa, CRISPR activation; MLV, murine leukemia virus; YFV, yellow fever virus; IAV, influenza A virus; MNoV, murine norovirus; ZIKV, Zika virus; VSVg, vesicular stomatitis virus glycoprotein.

### 4.2. Investigation of Fundamental Mechanisms of Antiviral Immunity

CRISPR-mediated inactivation of genes allows the investigation of antiviral mechanisms of target genes, assessing of immune signaling pathways, and the elucidation of mechanisms regulating host immunity. The most potent antiviral genes can be used as therapeutic targets for activation with CRISPRa tools. In addition, knocking down specific genes can aid the study of viral immune evasion and antiviral mechanisms of immune-targeting drugs. CRISPR-mediated gene inactivation has been used to examine genes involved in the most important immune pathways, including Toll-like receptor pathways, RIG-like receptor pathway, IFN signaling, IL-1β/caspase-1 pathway, STING pathway, ISG effector genes, and others (Table 3).

CRISPR-activation screens have been conducted using models of ZIKV [191] and murine norovirus [190]. Huh7 cells are highly sensitive to ZIKV infection and its cytopathic effect. CRISPR activation of IFI6 and IFNL2 strongly protected cells from ZIKV-induced death, with ISG20 and HELZ2 factors relieving cytopathic effects less profoundly [191]. Similar workflow was used in HeLa cells after challenge with murine norovirus (MNoV^CW^3 and MNoV^CR^6 strains). sgRNAs recruiting CRISPR activation systems to several genes were enriched in surviving cells. Further experiments revealed that overexpressing the TRIM7 gene had the highest antiviral effect, efficiently inhibiting norovirus replication and preventing virus-associated cytopathy [190].

The CHIME approach was created to analyze adaptive immunity and investigate gene function in desired subpopulations of immune cells in vivo. CHIME allows target gene knockouts in different cell subpopulations involved in innate (dendritic cells, macrophages) and adaptive (B-cells, CD4+—or CD8+—T-cells) immune response. The cells are used in in vivo chimeric mouse models, and genes involved in subpopulation proliferation/differentiation or effector genes can be used as targets.

The CHIME approach necessitates extracting bone marrow stem cells from transgenic Cas9-expressing mice and transducing these cells with lentivirus expressing sgRNA and a fluorescent reporter. Then, transduced cells are used to reconstitute the bone marrow in irradiated recipient mice. The resulting chimeric animals can be used for viral or tumor challenge, after which the subpopulation of immune cells can be investigated using flow cytometry. In addition to individually knocking out genes, this approach can also be used in a screening format with sgRNA libraries. The main advantage of CHIME is the absence of alterations in the natural development and maturation of immune cells with target genes knocked out by CRISPR [192].

### 4.3. Investigation of Signaling Pathways Involved in Antiviral Response

The interferon system is the main component of the innate antiviral immune response, and IFN types I and III play a major role in viral restriction. IFN signaling is induced by the recognition of viral patterns by sensors (Toll-like receptors, RIG-I-like receptors, DNA sensors) followed by the activation of adaptor proteins and subsequent activation of IRF3/7 transcriptional factors [193]. IRF3 and IRF7 bind to promoters of IFN genes and activate their transcription and secretion. Molecules of IFN I or III interact with IFN receptors and activate the IFN receptor signaling cascade. IFN signal transduction is induced by adaptor kinases that activate STAT transcription factors. STAT proteins translocate to the nucleus, form complexes with IRF9, and activate ISG expression. ISG expression is activated by binding of the above-mentioned complex to IFN-stimulated response elements in ISG promoter regions. ISG perform a wide spectrum of antiviral functions, including direct antiviral effects, metabolism regulation, and enhancing antiviral signaling [74]. CRISPR–Cas systems allow the inactivation of factors involved in different antiviral signaling pathways and are thus priceless molecular tools for investigating intracellular antiviral responses.

As mentioned in previous sections, DNA recognition in the cytoplasm occurs via the STING pathway. DNA sensors such as cGAS, IFI16, and Ku70/80 recognize DNA, including viral DNA, mitochondrial DNA, and nuclear DNA. Recognition results in signal transduction on the common adaptor STING protein, which induces IFN secretion in an IRF3-dependent manner. AIM2 DNA sensor stimulates inflammation by activating caspase-1 and mediating the cleavage of pro-IL-1β that results in the secretion of mature pro-inflammatory IL-1β [194]. Knocking out different STING pathway components using CRISPR–Cas results in impaired IFN secretion and increased replication of various DNA viruses, including herpesviruses, VACV, and others. Inactivating cGAS or STING in monocyte-derived dendritic cells and macrophages reduces IFN-β secretion after infection with DNA viruses (HCMV and VACV) but not with an RNA virus (VSV) [195]. Analogously, knocking out STING in HUVEC cells results in increased viral titers after CMV infection [196].

Using CRISPR–Cas, Sui et al. (2017) demonstrated the importance of STING in inducing type III IFN response after the recognition of exogenic DNA by Ku70. Depleting STING also resulted in decreased IFN-λ1 secretion after infection with HSV-2 [197]. Gray et al. (2016) showed that knocking out cGAS and STING repressed STING response and ISG activation after CMV infection. At the same time, inactivating another STING-dependent sensor, IFI16, did not affect the IFN response to CMV in cell models [198]. Diner et al. (2016) showed that IFI16 induces cytokine secretion after HSV infection via a STING/TBK-1/IRF-3-independent mechanism. Activating IFI16 leads to the direct transcriptional repression of HSV-1 and reduces viral titers, but this restriction can be antagonized by HSV-1 ICP0 ubiquitin ligase. In comparison, the cGAS sensor activates cytokine secretion through a STING-dependent pathway. In addition, during HSV infection, cGAS, but not IFI16-dependent DNA sensing, induces apoptosis [199].

Knocking out three OAS proteins (OAS1, OAS2, and OAS3) and the downstream effector protein RNAse L shows that OAS3 is a major factor providing 2′,5′-oligoadenylate synthesis for RNAse L activation, while the role of OAS1 and OAS2 in this process is negligible. OAS3-deficient and RNAse L-deficient cells had more evident replication of Sin Nombre virus (SINV), WNV, IAV, and VACV, and the titers of these viruses in OAS3-KO and RNAse L-knock-out cells were increased [200].

As RIG-I/MAVS is an important immune pathway during infection with RNA viruses, inactivating RIG-I leads to impaired response to these pathogens. Li et al. (2018) demonstrated decreased IFN-β secretion and ISG activation in RIG-I knockout cells after infection with Sendai virus and Seneca Valley virus, as well as increased Seneca Valley virus mRNA and protein levels [201]. CRISPR-mediated knockout confirmed RIG-I, but not MDA5, as the main sensor recognizing ZIKV RNA. RIG-mediated signaling leads to induction of ISGs after ZIKV infection. In the absence of RIG-I, ISG expression is diminished, and ZIKV-infected cells undergo apoptosis due to increased viral replication in cells. ZIKV NS5 can partially counteract RIG-I mediated activation of the IFN system through RIG-I inhibition [202].

CRISPR–Cas tools have been used to validate and characterize the main components of the IFN pathways. IFNAR1/IFNAR2 proteins (components of IFN I receptor) have been demonstrated to be required for STAT phosphorylation and the IFN I signaling cascade, as their depletion results in decreased ISG expression and increased viral replication after infection [203,204]. Knocking out STAT2 completely abolishes type I IFN-induced antiviral activity toward VSV and HCV, whereas STAT1 inactivation only partially inhibited these viruses [204,205]. In addition, STAT2 knockout cells are partially protected against encephalomyocarditis virus after IFN I treatment, which is an effect that is IRF1-dependent [204]. At the same time, type III IFN signaling is STAT1-dependent, as STAT1 inactivation completely suppresses IFN III antiviral activity toward HCV [205]. Knocking out STAT3 or STAT6 has no evident effect on IFN I/III antiviral activity [204,205]. Downstream IFN-related factor IRF9 is also a necessary component of the ISG activation complex, and the absence of IRF9 leads to the blockade of ISG activation after viral infection [205,206]. Several ISGs can be induced after viral infection in an IFN-independent, IRF3-dependent manner. Antiviral factors IFIT1, IFIT2, IFIT3, CXCL10, Mx1, Mx2, and ISG15 are induced by the IRF3 pathway during infection with CMV and other viruses [207].

### 4.4. Regulation of Antiviral Immunity

Regulation of antiviral immunity requires the activation of restriction factors after viral pattern recognition and silencing of the immune response after pathogen clearance. Disturbances in immune regulation can abrogate viral suppression or induce autoimmune tissue lesions. Therefore, investigating innate immunity regulation is critical. Ubiquitination is an important post-translational modification of cellular proteins and a significant mechanism of regulating immune pathways [208]. The function of different DUB family deubiquitinating enzymes in immune regulation was investigated by Liu et al. (2018) using CRISPR–Cas mediated knockout and viral challenge [209]. The group identified the important role of ubiquitination in innate immunity regulation and demonstrated negative feedback regulation loops for STING-mediated ubiquitination of an IFI16 sensor in an HSV-1 cell model. The STING-mediated ubiquitination of IFI16 led to the degradation of excess IFI16 molecules in proteasomes and prevented over-activation of the IFN I pathway [210]. E3 ubiquitin ligase TRIM41 is necessary for activating cGAS activity by monoubiquitination, and depleting this factor resulted in decreased IFN production and increased HSV-1 titers after infection [211].

Knocking out TTLL12 (Tubulin Tyrosine Ligase Like 12) permitted investigations of its role in response to RNA viruses and RIG-I pathway regulation. The main mechanism of TTLL12-induced RIG-I pathway inhibition is direct binding to VISA/MAVS adaptor proteins to prevent their interaction with TBK1 and IKKε [212]. Depleting the elongation initiation factor 4E binding protein (4E-BP1) leads to increased expression of IRF7 factor (main transcriptional factor involved in IFN gene induction). The upregulation of IRF7 resulted in increased type I IFN expression and enhanced antiviral response to VSV in a cell model [213].

Since DNA sensors can recognize host nuclear and mitochondrial DNA, the factors involved in clearing host DNA from the cytoplasm play an important role in regulating the basal activity of DNA sensors [214]. Suppressing these factors leads to more prominent sensor activation, increased ISG expression, and improved resistance to viral infections. Inactivating Banf1 in cells leads to increased accumulation of host DNA in the cytoplasm, increased ISG expression mediated by cGAS/STING, and increased susceptibility to viral infection, suggesting Banf1 as a factor that regulates basal ISG expression [215]. Loss of Cogesin complex factor STAG2 results in accumulation of host DNA fragments in the cytoplasm and in cGAS/STING-mediated induction of IFN I/III response. STAG2-deficient cells also have diminished levels of viral RNA after infection with various RNA viruses, including rotavirus, IAV, CHIKV, and VSV [216]. Using CRISPR–Cas-mediated gene inactivation, Kumar et al. (2018) demonstrated that TREX1 exonuclease is a main factor of HIV immunogenicity: knocking out TREX1 increases levels of HIV’s incomplete reverse transcription products, which are cGAS ligands. Increased TREX1 expression, on the other hand, results in the degradation of these partial genomes and abrogates ISG expression. However, TREX1 does not degrade full-length HIV DNA genomes, which are protected from degradation by proteins of the pre-integration complex [217].

### 4.5. Investigating Viral Immune Evasion Mechanisms

Although IFN I/III have broad antiviral activity, different viruses have evolved mechanisms of immune evasion to counteract host immune surveillance and to increase replication after infection. CRISPR–Cas-mediated inactivation of ISGs has been exploited to study several such mechanisms.

RNAse L is activated by 2′,5′-oligoadenylates after infection with RNA viruses, and its activation results in cleavage of viral RNA genomes, as well as of host rRNA and mRNA. Using CRISPR knockout of RNAse L, Whelan et al. (2019) demonstrated decreased levels of ZIKV RNA genomes after infection. Surprisingly, titers of viral particles in RNAse L knockout cell were similar to wild-type controls or even slightly higher. A possible mechanism by which ZIKV escapes RNAse L-mediated cleavage is by forming replication factories in invaginations in the membrane of the endoplasmic reticulum during early stages of infection. ZIKV genomes in such replication factories are resistant to RNAse L cleavage [218]. This mechanism is unique to ZIKV, as DENV, another flavivirus, also generates replication factories but is not resistant to RNAse L-mediated cleavage [218].

TLR signaling is an important part of IFN induction, so viruses have evolved mechanisms to block it. For instance, SIAH1 E3 ubiquitin ligase is overproduced during DENV infection; SIAH1 binds to the MyD88 adaptor protein of the TLR pathway, inducing its degradation and reducing antiviral IFN response. Schafer et al. used CRISPR–Cas to investigate this mechanism. The transient knockdown of SIAH1 reconstitutes high-level IFN response and decreases DENV replication. However, CRISPR-mediated knockout of MyD88 abrogates anti-DENV IFN response and results in an increase in DENV replication [219].

USP18 ubiquitin-specific proteinase negatively regulates the JAK/STAT arm of IFN I signaling cascade. HIV induces USP18 expression to suppress activation of the IFN pathway and increase viral replication. Inactivating USP18 using CRISPR–Cas led to increased IFN I signaling and reduced HIV viral loads [220]. Additionally, knocking out USP18 restores the antiviral activity of p21, which supports the amount of active SAMHD1 (HIV host restriction factor) [221].

Adaptor kinases are target proteins for viral evasion as well. It was demonstrated that the HCMV gene U_L_26 blocks the NF-κB pro-inflammatory response. CRISPR-knockout experiments revealed that this effect is mediated by inhibition of IKKβ kinase phosphorylation, which is the central kinase in the NF-κB pathway [222].

As CRISPR-mediated cleavage can introduce mutations into miRNA precursor genomic sequences, this molecular tool can be used to study the role of miRNA in antiviral immunity. HIV increases miRNA-146a and depletes the crucial immune adaptor kinases TRAF6 and IRAK1, inhibiting NF-κB pro-inflammatory response. Cas-mediated abrogation of miR-146a restored the activity of the NF-κB pathway, ablated HIV-1 replication, and proviral reactivation [223].

Inactivating effector ISGs using CRISPR–Cas can be applied to investigate the roles and mechanisms of these genes in restricting particular viruses. Using CRISPR, Dufrasne et al. (2016) investigated the antagonism between HIV Env protein and BST-2 host restriction factor. Mutation N659D in Env protein restored BST-2 activity and reduced HIV replication. Knockout of BST-2 resulted in active replication of the Env mutant HIV virus [224].

Hahn et al. (2016) investigated the role of individual components of the ND10 complex in inhibiting gamma-herpesvirus rhesus monkey rhadinovirus (RRV). Using CRISPR-mediated knockout of ND10 components, the authors showed that SP100 and PML (TRIM19) proteins of the complex are degraded by viral FGARAT homolog ORF75. Nevertheless, knocking out these factors only slightly increased viral replication. At the same time, inactivating DAXX and, to a lesser degree ATRX, increased infection with RRV. Thus, RRV does not completely deplete ND10 function, as DAXX protein is still able to restrict its replication [225].

HCMV infection activates the AIM2/caspase-1/IL-1β pro-inflammatory pathway. Botto et al. (2019) demonstrated that IFI16 and NLRP3, which are sensors of the caspase-1/IL-1β pathway, do not contribute to the secretion of IL-1β during HCMV infection [226]. However, AIM2 is required for this process, as its inactivation prevents IL-1β secretion after infection. cGAS/STING pathway activation contributes to increased IL-1β secretion after HCMV infection through the upregulation of AIM2. At the same time, HCMV actively attenuates the expression and secretion of mature IL-1β. The main mechanism by which HCMV evades the immune system is via the viral IE86 protein, which prevents NF-κB activity and induces pro-IL-1β degradation [226].

### 4.6. ISG Antiviral Action

To study how ISGs inhibit various viruses, effector genes can be knocked out in cells subsequently infected with the virus in question, with or without additional cytokine treatment. Thus, Xu et al. (2018) demonstrated an important role of MxB in restricting wild-type HIV but not VSVg-pseudotyped HIV. Additionally, the group showed that mutations in the viral capsid could make the virus resistant against MxB protein [227]. SAMHD1 restricts HIV infection mainly by decreasing intracellular dNTP and acts as a cell cycle-regulating factor and apoptosis inducer, also inhibiting HIV [228]. eIF4F initiation factor complex inhibits rotavirus infection by regulating intracellular IRF1 and IRF7 levels, and it can be negatively regulated by PDCD4 factor to increase rotavirus replication [229]. LXR factor mediates the inhibition of HSV-1 replication in a cell model; LXR activation depends on the function of CH25H via its main product, 25HC [230].

ISG15 has been demonstrated to be an important viral hemorrhagic septicemia virus inhibitor in a cell model, while Mx1 had no impact on viral infection [231]. APOBEC3A and APOBEC3B restrict HBV cccDNA pool replenishment, as CRISPR-mediated suppression of these factors did not significantly increase the level of viral transcripts in cells, but it did elevate levels of HBV cccDNA [232]. A complete list of antiviral factors investigated by CRISPR and the associated viruses is presented in Table 4.

The SARS-CoV-2 pandemic has claimed the lives of >3.5 million people. Despite clinical investigation of several antivirals, including FDA-approved drugs, no compounds have been effective in eliminating this virus. Therefore, activating antiviral immunity can be a therapeutic option for patients with COVID-19. Current investigations indicate that SARS-CoV-2 weakly induces IFN and ISGs [233]. For this reason, applying exogenous IFNs is a possible therapeutic option. Recent in vitro and clinical studies describe the efficient repression of SARS-CoV-2 infection after IFN treatment, if treatment is administered early. Zang et al. (2020) used a lentiviral library encoding 57 ISGs in a VSV-SARS-CoV-2 pseudovirus cell model to identify IFITM2, IFITM3, and CH25H genes as potent anti-SARS-CoV-2 factors, and CRISPR-mediated knockout of CH25H was used to validate the screening data. Adding 25HC to the cells restricted SARS-CoV-2 replication. A possible mechanism of 25HC’s antiviral action is its accumulation in late endosomes and the resulting alteration of cholesterol levels, leading to reduced SARS-CoV-2 S protein-mediated fusion [233].

Detailed investigation of several antiviral genes, including localization, intracellular trafficking, metabolism, and other processes can be difficult due to the absence of specific antibodies. CRISPR–Cas mediated homologous recombination permits the introduction of specific tags into gene-encoding sequences without disturbing gene function. The main advantage of CRISPR-mediating tag introduction over using exogenic vectors is the ability to study gene function in a more natural context without significant changes in gene expression. The applicability of this approach was demonstrated in an HIV model in which a hemagglutinin tag was added to the SERINC5 gene sequence [234]. Alternatively, a tagged gene can be reconstituted in cells using a lentiviral vector after CRISPR-mediated knockout. This method was used to generate a cell line with GFP-labeled TAP proteins [235]; an important component of the antigen-presenting apparatus, these proteins transport antigens from the cytoplasm to the endoplasmic reticulum for association with MHC-I molecules [236]. Depleting TAP activity reduced the amount of MHC I-peptide complexes on the surface of antigen-presenting cells. Defects in antigen presentation in turn abrogate T-cell-mediated adaptive immunity.

CRISPR–Cas systems can also be used to determine potential therapeutic targets of antiviral immunotropic drugs. Knocking out potential drug targets abrogates the antiviral effect of the drug of interest. This approach was used to investigate the G10 compound using different cellular models of infections with alphaviruses. G10 activates STING-dependent IFN secretion, inhibiting the replication of various viruses including CHIKV, VEEV, and SINV. MAVS inactivation had no significant effect on G10 antiviral activity [237]. In another study, R848 TLR7/8 agonist was demonstrated to mediate anti-ZIKV activity by activating the viperin ISG. Cas-mediated depletion of this factor reduced the antiviral effect of this compound by nearly two-thirds [238].

**Table 4:** Investigation of innate antiviral immunity using CRISPR–Cas technology.

Purpose of Investigation	Model Virus	CRISPR Target Genes	Signaling Pathways	Ref.
Signaling pathways	CMV	cGAS, IFI16, STING	STING pathway	[198]
	CMV, MVA, VSV	cGAS, IFI16, STING	STING pathway	[195]
	HSV-2	STING	STING pathway	[197]
	CMV	STING	cGAS/STING	[196]
	WNV, IAV, VACV, SINV	OAS1, OAS2, OAS3, RNAse L	OAS signaling	[200]
	SeV, SVV	RIG-I	RIG-I/MAVS	[201]
	VSV, EMCV	IFNAR1, IFNAR2, STAT1, STAT2, IRF1, STAT3	IFN receptor signaling	[204]
	IAV	IFNAR2	IFN receptor signaling	[203]
	HCV	STAT1, STAT2, IRF9, STAT3, STAT6	IFN receptor signaling	[205]
	VHSV	IRF9	IFN receptor signaling	[206]
	CMV	IRF3	ISG induction	[207]
Signaling pathways, ISG antiviral activity	HSV-1	IFI16, STING, cGAS, TRIM19	STING pathway,	[199]
			caspase-3 pathway,	
			effector ISGs	
Signaling pathwaysViral evasion	CMV	ASC, CASP1, AIM2, IFI16, NLRP3, cGAS, STING, IL-1β, GSDMD	STING pathway,	[226]
			IL-1β/caspase-1 pathway	
	ZIKV	RIG-I, MDA5, IFNAR	RIG-I/MDA5/MAVS	[202]
			IFN receptor signaling	
Immunity regulation	VSV, HSV-1, SeV	DUB family enzymes	RIG-I/MAVS	[209]
			STING pathway	
	SeV	TTLL12	RIG-I/MAVS	[212]
	WNV, SINV-EEEV, VSV, HSV-1	*Banf*, *cGAS*, *STING*, *Irf3*, and other STING pathway genes	STING pathway	[215]
	HSV-1	IFI16, STING	STING pathway	[210]
	RV, CHIKV, IAV, VSV	STAG2, STAT1, STING	cGAS/STING	[216]
	HIV-1	TREX1, cGAS	cGAS/STING	[217]
	HSV-1	TRIM41	cGAS/STING	[211]
	VSV	4E-BP1	TLR signaling IFN receptor signaling	[213]
Viral evasion	ZIKV, DENV, SINV	OAS3, RNAseL, STAT1, STAT2, MAVS	OAS signaling	[218]
			IFN receptor signaling	
			RIG-I/MAVS	
	DENV	MyD88	TLR signaling	[219]
	HIV-1	USP18	IFN receptor signaling	[220]
	CMV	ISG15, IKKα, IKKβ	NF-κB pathway	[222]
	HIV-1	miR-146a precursor sequence	NF-κB pathway	[223]
	HIV-2	BST-2	Effector ISGs	[224]
	HIV-1	USP18	Effector ISGs	[221]
	IAV	p53	Effector ISGs	[239]
	RRV, KSHV	TRIM19, SP100, DAXX, ATRX	Effector ISGs	[225]
ISG antiviral activityViral evasion	HIV-1	STAT1, MxB and 53 other ISGs	ISG induction	[227]
ISG antiviral activity	HIV-1	SAMHD1	Effector ISGs	[228]
	RV	eIF4A, eIF4E, eIF4G	Effector ISGs	[229]
	HSV-1	LXRα, LXRβ, CH25H, SULT2B1	Effector ISGs	[230]
	SARS-CoV-2	CH25H	Effector ISGs	[233]
	VHSV	Mx1, ISG15	Effector ISGs	[231]
	HBV	APOBEC3A, APOBEC3B	Effector ISGs	[232]
	HBV	MOV10	Effector ISGs	[240]
	HPV16	SAMHD1	Effector ISGs	[241]
	HIV-1	SERINC5 (KO/iHA Knock-in)	Effector ISGs	[234]
	SHIV	IFITM1, IFITM3, IFITM3A	Effector ISGs	[242]
	IAV, ZIKV, VEEV, YFV, ONNV, WNV, VSV, DENV	IFITM1, IFITM2, IFITM3	Effector ISGs	[243]
	VACV mutant	SAMD9, WDR6	Effector ISGs	[244]
	Poxviruses	mSAMD9L, hSAMD9L	Effector ISGs	[245]
	HSV-1	IFI16	Effector ISGs	[246]
	ZIKV	Viperin	Effector ISGs	[247]
Drug antiviral mechanisms	CHIKV, VEEV, SINV	IRF3, STAT1, MAVS, STING	STING pathway	[237]
			JAK/STAT	
			RIG-I/MAVS	
	ZIKV	Viperin	TLR signaling	[238]
			Effector ISGs	

Abbreviations: VSV, vesicular stomatitis virus; HSV, herpes simplex virus; SeV, Sendai virus; WNV, West Nile virus; SINV-EEEV, chimeric eastern equine encephalitis virus; EMCV, encephalomyocarditis virus; IAV, influenza A virus; HCV, hepatitis C virus; VHSV, viral hemorrhagic septicemia virus; CMV, cytomegalovirus; MVA, modified vaccinia virus Ankara; RV, rotavirus; CHIKV, Chikungunya virus; VACV, vaccinia virus; SINV, Sindbis virus; SVV, Seneca Valley virus; ZIKV, Zika virus; DENV, Dengue virus; HIV, human immunodeficiency virus; RRV, rhesus monkey rhadinovirus; KSHV, Kaposi sarcoma herpesvirus; HPV, human papillomavirus; SHIV, simian immunodeficiency/human immunodeficiency chimeric virus; VEEV, Venezuelan encephalitis virus; YFV, yellow fever virus; ONNV, o’nyong’nyong virus; OAS, oligoadenylate synthetases.

### 4.7. Developing Antiviral Approaches

Cytokine-based therapy is recommended for treating several viral infections [248,249,250]. However, since viruses have different mechanisms of immune evasion and establishing persistence, cytokines are not always effective. Most immune evasion mechanisms focus on the evasion of immune recognition or receptor signaling blockade. CRISPR-activation technology allows the direct transcription of host restriction factors without involving signaling pathways. For this reason, using CRISPRa to manipulate antiviral genes is a perspective therapeutic to combat viral infections.

Several such genes were activated by CRISPRa in in vitro models of viral infection. In particular, effective HIV depletion was demonstrated during the activation of genes with strong antiretroviral activity, including APOBEC3B and APOBEC3G. The effectiveness of the APOBEC antiviral effect can be partially diminished by the viral antigen vif [251]. Zhang et al. (2019) used CRISPRa to induce BST-2/tetherin, which is one of the most potently antiretroviral ISGs. After BST-2 activation, levels of HIV p24 in culture media decreased even in the presence of BST-2 viral antagonist Vpu. Instead, large numbers of viral particles remained tethered to the surface of cells hyperexpressing BST-2 protein; such tethering is the main antiviral mechanism of BST-2 [252].

One potential method for completely eliminating HBV cccDNA, the virus’s persistent genome, is activation of APOBEC factors. Similarly, overexpressed AID efficiently deaminates HBV cccDNA and purges viral DNA out from the nuclei of infected cells [253]. It should be noted that the activation of APOBEC3 factors by dCas-based approaches efficiently degrades episome-like genomes that are natural targets of APOBEC proteins but does not efficiently deaminate viral genomes integrated into human chromosomes [254]. Transient gene activation is an important advantage of CRISPRa, as prolonged overexpression of host restriction factors can induce pathologic states, including toxicity and malignant transformation [255]. Potentially, CRISPRa of most potent viral restriction factors can be used to treat a wide spectrum of viral infections.

CRISPR-mediated gene editing can be used to increase the activity of different antiviral ISGs. For example, the D128K mutation in the APOBEC3G gene allows it to escape counter-action by HIV vif antigen [256]. R332G/R335G mutations in TRIM5α significantly increase its capacity to inhibit HIV [257]. Dufour et al. (2018) used CRISPR-mediated homologous recombination to introduce R332G/R335G mutations into TRIM5α in human cells. The main limitations of this method are its low editing efficiency and high occurrence of unwanted indels [258]. Applying more precise CRISPR technologies for pinpoint editing (e.g., BE) may improve the efficiency and specificity of editing host antiviral genes.

CRISPRa can be also be used to stimulate the adaptive immune response. The viral latency state is characterized by viral persistence with minimal production of viral antigens, and infected cells with latent virus are not recognized by antigen-specific immune cells. For HIV therapy, a shock-and-kill approach can be used. This method is based on reactivating latent HIV provirus in combination with traditional antiretroviral therapy. Then, the overexpression of HIV antigens permits immune elimination of infected cells or destruction of infected cells directly due to viral antigen cytotoxicity. CRISPRa systems based on SunTag-VP64, VPR, or SAM have been used to successfully reactivate HIV provirus [259,260,261,262]. The main challenge in CRISPRa-based shock-and-kill HIV therapy approach is delivering the activation system to infected cells.

In summary, developing CRISPR-based approaches for modulating antiviral immunity allows the identification of new therapeutic targets in high-throughput models. The most potent factors identified by such screens can be subsequently activated using CRISPRa methods as a therapeutic approach to treat various viral infections. CRISPR-based screening requires valid and informative in vitro models of viral replication and implementing such models will boost CRISPR-based discovery of new antiviral factors. CRISPR-mediated knockout of desired genes is a potent molecular tool for investigating fundamental aspects in both innate and adaptive immunity, including mechanisms of action, regulation, viral immune evasion, and others.

## 5. Modulating Epitranscriptomics in Viral Infections

Epitranscriptomics is a dynamically developing research field. The main RNA epigenetic mark is methyl-6-adenosine (m6A) [263]. While its function is not completely understood, its role in different RNA metabolism processes has been established, including translation, degradation, splicing, export, and folding [264]. The regulation of m6A deposition and functions is mediated by three classes of enzymes: m6A writers, m6a erasers, and m6A readers [265]. Writers are part of the RNA methyltransferase complex, which catalyzes the methylation of adenosine residues on RNA and establishes m6A markers (for example, METTL3, METTL14, and other proteins). Erasers perform the opposite function, removing m6A residues. Among m6A erasers are ALKBH5 and FTO proteins. m6A readers bind m6A residues on RNA and enhance translation, induce degradation, or perform other functions. Examples of m6A readers are YTHDC1/2 and YTHDF1/2/3 proteins (m6A metabolism reviewed in [266,267]).

To date, m6A metabolism is known to significantly impact the life cycle of pathogenic viruses (including HIV, EV71, IAV, KSHV, HBV, HCV, HPV, ZIKV, and EBV). In viral biology, m6A can regulate viral replication and/or viral RNA stability, latency state, cell cycle, and other functions. m6A is also involved in the progression of virus-related tumors (including HBV- and HCV-related hepatocellular carcinoma and HPV-related cervical carcinoma) (reviewed in [268]).

The participation of m6A in regulating antiviral immunity has been described as well [269]. hnRNPA2B1, a recently identified nuclear sensor of viral DNA, amplifies type I IFN response after HSV-1 infection by inducing cGAS, IFI16, and STING proteins via m6A-dependent binding of hnRNPA2B1 to corresponding mRNAs; this subsequently increases the nuclear export and translation of these proteins [270]. Using a model of human metapneumovirus, Lu et al. (2020) demonstrated that m6A deposition on viral RNA reduces its recognition by RIG-I RNA sensors and decreases the activation of IFN secretion. Viral RNA may be recognized as self RNA by RIG-I sensors after m6A deposition, allowing immune evasion [271]. Similarly, Chen et al. (2019) reported that m6A can serve as a marker of self RNA to prevent the activation of sensors such as RIG-I, MDA5, OAS, OASL, and PKR [272]. Additionally, Winkler et al. (2019) demonstrated that IFNB mRNA can be targeted for methylation by m6A deposition, blocking the IFN response. Depleting m6A writers and readers activates the IFN response and depletes viral propagation in models of different viral infections, including CMV, IAV, adenovirus, and VSV. Moreover, CMV infection leads to an increased expression of m6A writers and readers, potentially increasing the degradation of IFN mRNA to allow immune evasion [269]. At the same time, DDX46 factor has been demonstrated to mobilize the m6A eraser ALKBH5 to induce the retention of Mavs, Traf3, and Traf6 mRNAs in the nucleus and prevent their translation. Thus, DDX46 negatively regulates IFN response by inducing m6A depletion (not deposition) on mRNA of immune factors.

Investigations of epitranscriptomics were initially based on CRISPR–Cas mediated knockout of m6A modulators (writers, readers, or erasers) [269]. Such an approach only increased or decreased the overall levels of m6A methylation, but some m6A-related effects have been shown to be dependent on RNA localization [273]. Creating systems for site-specific m6A methylation/demethylation has become a new goal of epitranscriptomics. Systems for RNA methylation are based on using a dCas9 protein fused to single-chain METTL14-METTL3 methyltransferase complex (dCas9-based writer). The target site for RNA methylation is determined by sgRNA and short PAM-containing antisense DNA oligonucleotide (PAMmer). PAMmer is necessary for the system to function, as Cas9-based proteins cannot bind RNA independently of it. m6A demethylation systems are dCas9 proteins fused to ALKBH5 or FTO m6A demethylation enzymes (dCas9-based erasers) [274]. Replacing the dCas9 protein in this system with a type VI dCas13 protein eliminated the necessity for PAMmers, because type VI proteins, including dCas13, are specific to ssRNA and can bind it independently. These modified m6A writer and eraser systems have become simpler and more convenient than dCas9-based systems [275].

## 6. Conclusions

Innate immunity is the first line of defense against infectious pathogens. Vertebrate hosts have evolved multiple mechanisms to restrict viral infection, suppress viral replication, and ultimately clear the virus from the body. However, under such selective pressure, viruses efficiently adapt to evade immunity and establish productive infection. Viruses can block the expression and/or function of various host proteins, become resilient to antiviral factors, or develop a stealth mode to avoid the recognition and triggering of immune responses. The interaction of viruses with infected cells is an area of active research that is promising not only for fundamental discoveries but also for translational studies and clinical practice.

Although potentially very promising, CRISPR–Cas approaches suffer from a number of limitations that may perturb their application in vivo, in clinical practice. First, the off-target activity of CRISPR–Cas systems is one of the most important challenges that is yet to be overcome despite the recent development of high-fidelity Cas variants. The generation of megabase-long indels, chromothripsis, etc. as a result of on-target or off-target nucleolytic cleavage of DNA may compromise the attempts to use Cas nucleases for therapeutic purposes [151,276]. Similar to Cas nucleases, the off-target activity of RNA-nucleases and RNA base editors was described. A reduction in the off-target activity of CRISPR-based approaches is possible by using evolved Cas proteins and functional domains with improved properties, orthologous nucleases, modified sgRNAs, or by introducing CRISPR–Cas as ribonucleoprotein complexes [277,278,279,280,281]. Second, Cas proteins are derived from bacteria and archaea, so that many enzymes are immunogenic and can be rapidly eliminated from the body by pre-existing immunity [282]. Among other approaches, the engineering of Cas proteins with immunosilenced or masked epitopes [283], and the application of Cas proteins from non-pathogenic organisms could become potential solutions for the safe, repeated administration of CRISPR–Cas in vivo. Last, but not least, is the issue of CRISPR–Cas in vivo targeted delivery, which is hampered by the large molecular weight of Cas proteins and dCas-based proteins as well as opportunities to deliver CRISPR–Cas as DNA, RNA, or in the form of ribonucleoprotein complexes. Pros and cons of these strategies are reviewed in [284].

To conclude, novel CRISPR and CRISPR-based tools are undoubtedly the most robust and efficient means to investigate virus–host interactions, identify and overcome mechanisms of viral immune evasion, and develop novel antiviral approaches. CRISPR–Cas has already revolutionized fundamental and translational research, as well as the field of molecular diagnostics. Clinical application of CRISPR–Cas techniques has recently provided promising early in vivo results to cure human genetic [285] and infectious diseases [286]. Further progress in this field will profoundly depend on the safety of CRISPR–Cas technologies for particular purposes and the effective delivery of gene editing complexes to the desired organs.

## Figures and Tables

**Table 2:** Comparison of most common CRISPRa, CRISPRi, and base editing systems.

System Effect	System	Number of Main Components	Relative Efficacy		Ref.
Activation	dCas9–VP64	2	↑		[161]
	dCas9–VP160/VP192	2	↑↑		[158]
	dCas9–p65/p65–HSF	2	↑/↑↑		[165]
	dCas9–p300	2	↑↑		[160]
	dCas9–VPR	2	↑↑↑		[163]
	dCas9–CBP	2	↑↑↑		[166]
	Scaffold	3	↑↑↑		[162]
	dCas9–SunTag–VP64	3	↑↑↑		[164]
	SPH (SunTag–p65–HSF1)	3	↑↑↑↑		[167]
	SAM	3	↑↑↑↑		[165]
Repression	dCas9–KRAB	2	↑↑		[168]
	dCas9–KRAB–MeCP	2	↑↑↑		[177]
	dCas9–EZH2	2	↑↑		[169]
**Base Editors**					
**System**		**Target**	**Base Change**	**Editing Window (nts from PAM)**	**Ref.**
APOBEC1–BE3		DNA	C→T/G→A	13-17	[178]
APOBEC1–BE4		DNA	C→T/G→A	13-17	[179]
APOBEC3A–BE3		DNA	C→T/G→A	13-17	[180]
BE–PLUS		DNA	C→T/G→A	7-17	[181]
CRISPR-X		DNA	C→T/G→A	−50 to +50 from PAM	[182]
ABE-7.10		DNA	A→G/T→C	14-17	[183]
REPAIR		RNA	A→I	-	[173]
RESCUE		RNA	A→I/C→U	-	[174]
CURE		RNA	C→U	-	[175]

## Data Availability

Not applicable.

## Abbreviations

HIV	Human immunodeficiency virus
HBV	Hepatitis B virus
HCV	Hepatitis C virus
HDV	Hepatitis D virus
NK	Natural killer cells
IFN	Interferon
ISGs	Interferon-stimulated genes
JAK/STAT	Janus kinase/signal transducers and activators of transcription
DSB	Double-strand breaks
PAMP	Pathogen-associated molecular patterns
PRR	Pattern recognition receptors
TLR	Toll-like receptors
ssRNA	Single-stranded RNA
dsRNA	Double-stranded RNA
RIG-I	Retinoic acid-inducible gene I
cGAMP	Cyclic GMP-AMP
cGAS	Cyclic GMP-AMP synthase
AIM2	Absent in melanoma 2
TIR	Toll/IL-1 receptor
TRIF	TIR domain-containing adaptor-inducer interferon-β
MAVS	Mitochondrial antiviral-signaling protein
ssDNA	Single-stranded DNA
STING	Stimulator of interferon genes
ALRs	AIM2-like receptors
ASC	Apoptosis-associated speck-like protein containing a CARD
25HC	25-hydrocholesterol
VSV	Vesicular stomatitis virus
HSV	Herpes simplex virus
EBOV	Ebola virus
ZIKV	Zika virus
NCOA7	Nuclear receptor coactivator 7
IAV	Influenza A virus
DENV	Dengue virus
WNV	West Nile virus
PKR	Protein kinase R
SLFN11	Schlafen 11
APOBEC	Apolipoprotein B mRNA-editing enzyme catalytic polypeptide-like
IFI16	Gamma-interferon-inducible protein 16
IFITM1	Interferon-induced transmembrane protein 1
OAS	Oligoadenylate synthetase
VACV	Vaccinia virus
HCMV	Human cytomegalovirus
KSHV	Kaposi’s sarcoma-associated herpesvirus
MV	Measles virus
LMP1	Latent membrane protein 1
YFV	Yellow fever virus
HDAC	Histone deacetylase
SINV	Sin Nombre virus
ONNV	o’nyong’nyong *virus*
SINV	Sindbis virus
SVV	Seneca Valley virus
VEEV	Venezuelan encephalitis virus
CHIKV	Chikungunya virus
RV	Rotavirus
MVA	Modified vaccinia virus Ankara
CMV	Cytomegalovirus
SINV-EEEV	chimeric eastern equine encephalitis virus
SeV	Sendai virus
m6A	methyl-6-adenosine
TTLL12	Tubulin Tyrosine Ligase Like 12
